# Critical fluctuations and slowing down of chaos

**DOI:** 10.1038/s41467-019-10040-3

**Published:** 2019-05-14

**Authors:** Moupriya Das, Jason R. Green

**Affiliations:** 1 Department of Chemistry, University of Massachusetts Boston, 100 Morrissey Boulevard, Boston, MA 02125 USA; 2 Department of Physics, University of Massachusetts Boston, 100 Morrissey Boulevard, Boston, MA 02125 USA; 3 Center for Quantum and Nonequilibrium Systems, University of Massachusetts Boston, 100 Morrissey Boulevard, Boston, MA 02125 USA; 4 Present Address: Max-Planck Institute for the Physics of Complex Systems, 01187 Dresden, Germany

**Keywords:** Molecular dynamics, Condensed-matter physics, Statistical physics, thermodynamics and nonlinear dynamics

## Abstract

Fluids cooled to the liquid–vapor critical point develop system-spanning fluctuations in density that transform their visual appearance. Despite a rich phenomenology, however, there is not currently an explanation of the mechanical instability in the molecular motion at this critical point. Here, we couple techniques from nonlinear dynamics and statistical physics to analyze the emergence of this singular state. Numerical simulations and analytical models show how the ordering mechanisms of critical dynamics are measurable through the hierarchy of spatiotemporal Lyapunov vectors. A subset of unstable vectors soften near the critical point, with a marked suppression in their characteristic exponents that reflects a weakened sensitivity to initial conditions. Finite-time fluctuations in these exponents exhibit sharply peaked dynamical timescales and power law signatures of the critical dynamics. Collectively, these results are symptomatic of a critical slowing down of chaos that sits at the root of our statistical understanding of the liquid–vapor critical point.

## Abstract (web summary)

It is well known that fluids become opaque at the liquid–vapor critical point, but a description of the underlying mechanical instability is still missing. Das and Green leverage nonlinear dynamics to quantify the role of chaos in the emergence of this critical phenomenon.

## Introduction

Fluctuations are sovereign in critical phenomena^1,2^. They rule fluids at the liquid–vapor critical point, a unique instability that punctuates the space of thermodynamic states^3^. First established experimentally by Andrews^4^, the liquid–gas critical point was given a molecular explanation shortly thereafter by van der Waals^5^. In van der Waals’ picture, now a paradigm in liquid state theory^6,7^, repulsive forces largely determine the structural arrangements of molecules in non-critical liquids, not the attractive forces. Near the liquid–vapor critical point, however, their roles reverse and the paradigm shifts^8^; dynamical fluctuations reach macroscopic magnitude and overrule molecular size, shape, and interactions in dictating bulk behavior. These fluctuations are generated by the nonlinear dynamics of classical critical fluids. Yet, the relationship between the microscopic instability of the dynamics and the thermodynamic singularity has never been entirely clear^9^.

While the field of critical phenomena continues to absorb increasingly diverse systems^10^, the basic phenomenology is firmly established^1^. Its taxonomy is built on scaling and universality, the similar behavior of dissimilar systems. Despite the early discovery of their critical points, fluids were somewhat resistant to classification^5^. Simulations^11^ and theory^12^ were, and continue to be, integral in providing mechanistic insights, the location of the critical point, and estimates of static critical exponents^13–16^. Through simulations, the classical atomistic dynamics of fluids are known to be chaotic^17^, a part of the machinery of nonlinear dynamics. Measuring deterministic chaos has given insights into the physical mechanisms of the jamming transition in granular materials^18^, self-organizing systems^19^, evaporating collections of nuclei^20,21^, and the phase changes of atomic clusters^22–25^. In addition, the dynamics of model spatially-extended systems have recently begun to collect into dynamic universality classes^26^. These findings are part of efforts to coalesce statistical physics and nonlinear dynamics, and they reinvigorate the question of how fluids, specifically the properties of their molecular dynamics, fit within this phenomenological architecture of critical phenomena.

At the liquid–vapor critical point, how do correlations in molecular positions overcome the destabilizing force of deterministic chaos in the molecular dynamics? Here, we resolve the instability of the molecular motion that generates this critical phenomenon. Because of the absence of long-range order—and an inability to make a small vibration approximation, as in solids, or a molecular randomness hypothesis, as in gases^27^—the dynamical instability in critical fluids has been largely grounded in purely statistical terms. However, critical correlations imply structural organization that is intrinsically opposed by a chaotic dynamics. Both numerical simulations and analytically tractable models show here that the critical dynamics carry signatures of this internal tension between order and chaos. We find that Lyapunov exponents^28^, a measure of chaos and dynamical instability, are minimal at the liquid–vapor critical point. Signatures also appear in the finite-size scaling of fluctuations in these observables. The fluctuations decay as a power law towards the thermodynamic limit with the slowest rate at the critical point. Overall, these results suggest this singular state is a limit of dynamical order that long-range correlations can impose on the dynamics.

## Results

### Dynamics of a simple fluid

**Fig. 1:**
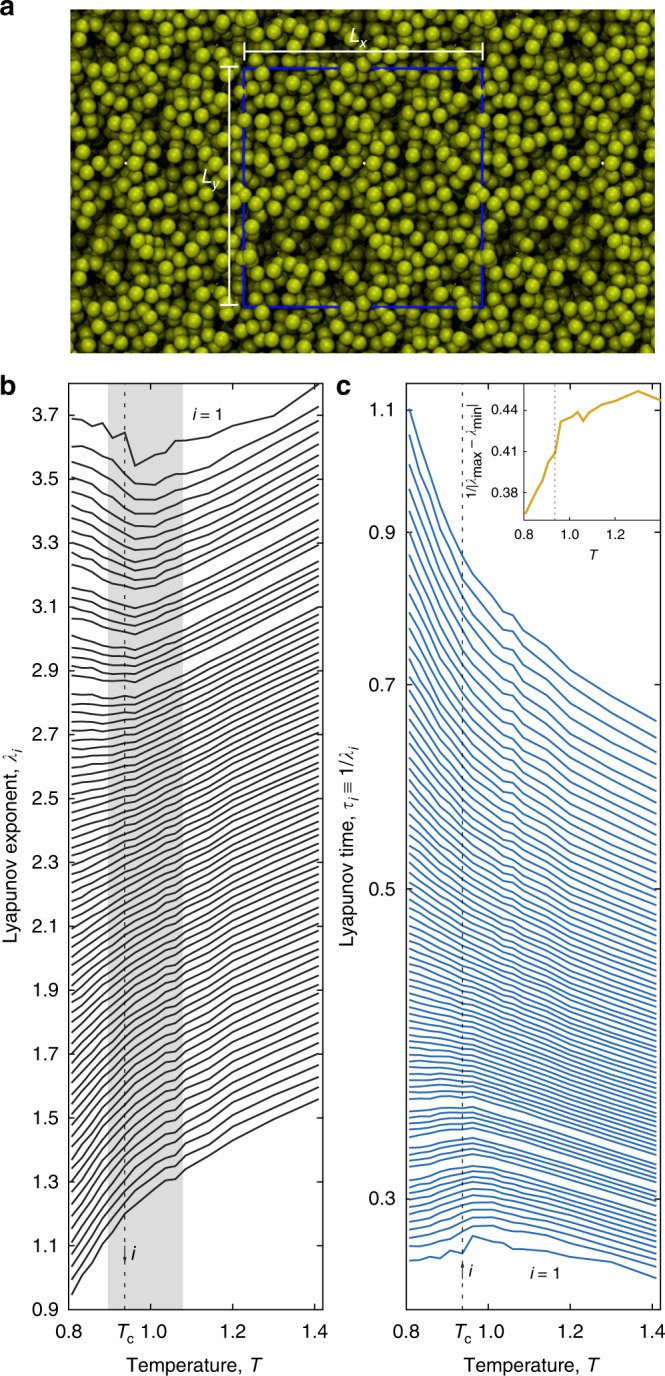
Slowing down the divergence of trajectories at the liquid–vapor critical point. **a** Cross-sectional snapshot of the critical fluid and the periodic boundaries of length *L* = *L*_*x*_ = *L*_*y*_ = *L*_*z*_. **b** Spectra of positive Lyapunov exponents *λ*_*i*_ and **c** Lyapunov times *τ*_*i*_ (log-linear scale) as a function of the mean kinetic temperature, *T*. Every 30 *λ*_*i*_ are shown for *N* = 1000 particles occupying a cubic simulation volume with a density *ρ* = *ρ*_c_ = 0.317. Unstable spatiotemporal vectors that correspond to more disordered motions (1/3*N* ≤ *i*/3*N* ≤ 0.18) have positive Lyapunov exponents (time) with a minimum (maximum). Vertical dashed lines mark the critical temperature, *T*_c_. Inset illustrates compression of spectrum through the critical point for data shown

To analyze the molecular dynamics at the liquid–vapor critical point, we numerically simulate a homogeneous, single-component, non-associated, equilibrium fluid (Fig. 1a) and apply techniques from nonlinear dynamics. The fluid consists of *N* molecules interacting pairwise through van der Waals forces, repelling (attracting) at distances of a (few) molecular diameter(s) according to the Lennard–Jones (LJ) potential^29^. As order parameters, we use the hierarchy of 6*N* spatiotemporal (Lyapunov) vectors. Each vector has an associated exponent, *λ*_*i*_ indexed *i* = 1,…,6*N*^28^ and in descending order, that measures the contribution of each vector to the global dynamics. Larger exponents indicate more unstable phase space directions^30^. We calculate the full Lyapunov spectrum at the critical density *ρ*_c_ and over a range of temperatures including the critical temperature, and we choose the energy density to fix the mean kinetic temperature, *T*. Each *λ*_*i*_ is a time-average of a 10^6^ time step trajectory.

### Suppression of chaos at the critical point

When approaching the liquid–vapor critical point from the supercritical regime, *T* > *T*_c_, the Lyapunov exponents decrease monotonically (Fig. 1). This decline represents a “slowing down” in the divergence of trajectories as *T* approaches *T*_c_ and accompanies the transient formation of spatial regions in the system that will separate into coexisting vapor and liquid phases below *T*_c_. What drives this decrease in the largest exponent, *λ*_1_, is a suppression of the fastest dynamical events^31^. Here, these events are molecular collisions that sample the repulsive part of the intermolecular potential. As the system “practices” phase separating, as Widom put it^5^, fewer of these high-energy collisions occur^32^.

Away from the critical point, the van der Waals picture is the foundation for the statistical-mechanics of liquids^6^. Perturbative treatments, for example, assume the structure of a dense, monatomic liquid resembles that of a hard sphere fluid and, to a first approximation, the attractive interactions have little effect on the liquid structure^6^. At the critical point, though, repulsive intermolecular forces play a subordinate role compared to critical fluctuations. While the van der Waals picture of fluids was only recently shown to extend to the Lyapunov exponents and their fluctuations^33^, it does not hold near the critical point^8^. A steady 7% decrease with temperature and the clear minimum near *T*_c_ in the first Lyapunov exponent are direct evidence that critical conditions inhibit the effect of repulsive forces on the dynamics, consistent with the breakdown of the van der Waals picture.

Also evident from Fig. 1 is a minimum in the largest Lyapunov exponent near the known^34^ critical point, (*T*_c_, *ρ*_c_). The minimum in this exponent, and the maximum in the Lyapunov time, 1/*λ*_1_, near *T*_c_ means the critical dynamics are predictable over longer timescales because initially similar configurations do not diverge as quickly (Fig. 1c). For the finite-size systems we simulate, the doubling of the Lyapunov time is in accord with the doubling of the characteristic time of the autocorrelations in the kinetic energy (Supplementary Note 1). A suppression of the dynamical instability in the critical regime aligns with the physical intuition that long-range correlations are at play near *T*_c_. However, for the finite-size systems we simulate, the dynamics are not entirely predictable at *T*_c_; the first exponent has a non-zero value through the temperature range including *T*_c_, which suggests the continuous liquid–vapor phase change remains chaotic throughout and into the coexistence region for finite-size systems. These chaotic critical dynamics differ from the jamming transition in granular materials, which seems to be a transition from a chaotic to a non-chaotic state^18^.

To more fully resolve dynamical instability across the critical point, we also calculated the full spectrum of Lyapunov exponents. From simulations of two and three-dimensional liquids, the shape of the spectrum depends on the kinetic temperature and density (Supplementary Fig. 2)^32,35,36^. Figure 1b shows the long-time Lyapunov spectrum at the critical density for temperatures spanning the critical temperature, *T*_c_. These data for *N* = 1000 converged to <1% and show all unstable vectors have exponents that decrease when approaching the critical point from above. But, only the most unstable vectors have exponents with minima at the critical point. Critical correlations appear to have the largest impact on the vectors with scaled index up to *i*/3*N* ≈ 0.18 (Supplementary Fig. 3). Exponents beyond this point decrease monotonically with temperature. The spectrum is also compressed at the critical point (Fig. 1c inset), meaning there is a weaker preference for trajectories to diverge in the direction of any given vector.

Many models and molecular simulation techniques have been used to probe the liquid–vapor critical point^37,38^. But, the long-time Lyapunov exponents in Fig. 1 are the first glimpse of its chaotic properties. These exponents are intensive observables in the liquid and supercritical phases, but there is a slight dependence of the spectrum on the *N* at *ρ*_c_ that affects the location of the minimum (Supplementary Figs. 4 and 5). A similar weak dependence of the leading Lyapunov exponent on *N* was found for a two-dimensional LJ fluid^39^. To support our numerical simulations and pinpoint the location of the minimum, we analyze two analytically tractable approximations to a model system. The Lyapunov exponents and the Lyapunov time *τ* at the critical temperature *T*_c_ depend strongly on the nature of the approximation.

Because the liquid–vapor critical point is generally considered to belong to the Ising universality class^14,16^, we consider a continuous analog of the Ising model. The model is one-dimensional system of *N* interacting, classical particles of mass *m* in a bistable potential. Two approximations of this model capture the features of the heat capacity at the liquid–vapor critical point: the singularity and the finite-jump discontinuity^8^. To analyze the effect of a diverging heat capacity on the Lyapunov exponents, we treat the average effects of the anharmonic term in the Hamiltonian^9^ by imposing a temperature-dependent weakening of the anharmonic restoring force on each particle. The Lyapunov exponents are given by

1 $$\lambda _ \pm (T) = \pm \sqrt {\frac{{\kappa _2}}{m}} \left( {\frac{{|T - T_{\mathrm{c}}|}}{{T_{\mathrm{c}}}}} \right)^{1/2}.$$

**Fig. 2:**
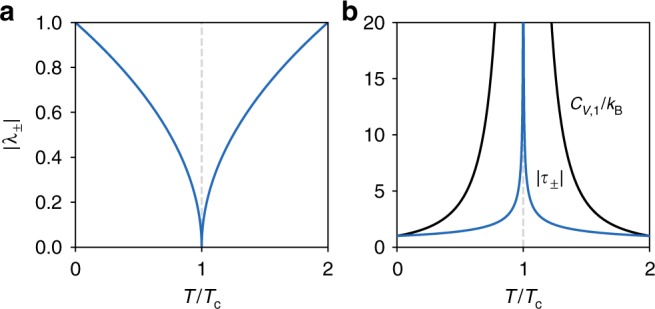
Analytical model of nonlinear oscillators with a dynamically stable critical point. At the critical temperature *T*_c_, **a** the *N* conjugate pairs of Lyapunov exponents, |*λ*_±_|, vanish and **b** the single-particle heat capacity, *C*_*V*,1_/*k*_B_, and Lyapunov time, |*τ*_±_| ≡ 1/|*λ*_±_| ∝ |*T*−*T*_c_|^−^^1/2^, diverge

There is a conjugate pair of exponents for each of the *N* particles. Approaching the critical point, the first Lyapunov vector will generate the instability that leads to the phase transition; the Lyapunov exponents become degenerate and their associated Lyapunov vectors become linearly dependent. From the last expression, we see that *λ*_±_ → 0 as *T* → *T*_c_. That is, all Lyapunov exponents vanish at the critical temperature according to the power law |*λ*_±_| ∝ |*T*−*T*_c_|^1/2^ (Fig. 2). The corresponding Lyapunov time diverges *τ*_±_ = 1/*λ*_±_ ∝ |*T*−*T*_c_|^−1/2^ at *T*_c_ and coincides with the divergence in the isochoric heat capacity.

Applying another (mean-field) approximation to the coupled-particle system, we can analyze the Lyapunov exponents when the heat capacity has a simple discontinuity and further confirm that the critical point is a limit of dynamical order (Supplementary Fig. 6)^40–42^. As an order parameter, we use the mean particle displacement. This order parameter is nonzero for *T*  < *T*_c_ and zero for *T* ≥ *T*_c_ (Supplementary Fig. 7). While the Lyapunov exponents are nonzero, they have a minimum at *T*_c_. The Lyapunov times have a maximum. The rate of change of the Lypaunov exponents and times with temperature have a finite-jump discontinuity at *T*_c_ mirroring the jump in the isochoric heat capacity, a typical feature of mean-field theories. It is well known that this jump in the heat capacity predicted by mean-field theory is embedded in a logarithmic divergence when fluctuations are included. Also known is that the exact scaling of observables at the critical point is strongly affected by statistical correlations in molecular positions.

Overall, this model of nonlinearly coupled particles gives evidence that the minima in the Lyapunov exponents are at *T*_c_ for pure fluids. The minima also support the sensitivity of the most unstable Lyapunov vectors to the critical dynamics and motivate a closer look at their finite-time fluctuations in our simulations.

### Suppression of approach to the thermodynamic limit

**Fig. 3:**
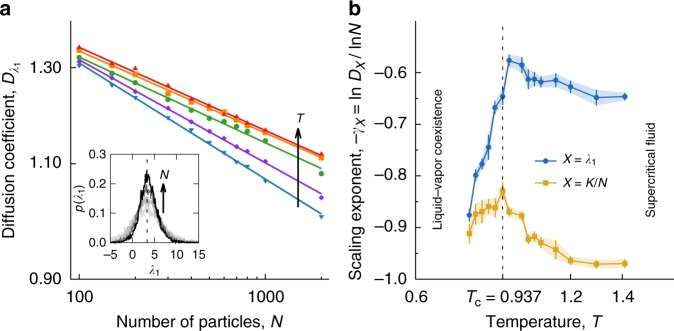
Finite-size scaling of fluctuations in dynamical observables. **a** Fluctuations in the kinetic energy and Lyapunov exponents decay with increasing number of particles (log–log): the diffusion coefficients follow a power law $D_X(N,T)\sim N^{ - \gamma _X(T)}$ for 11 system sizes from *N* = 100 to 2000. Data shown for *λ*_1_(*t*). The corresponding probability distributions also concentrate around the long-time value *λ*_1_ (Inset: first Lyapunov exponent at *ρ* = *ρ*_c_ = 0.317 and *T* = *T*_c_ = 0.937). **b** Wandering exponents, *γ*_*X*_(*T*), of the first finite-time Lyapunov exponent and the kinetic energy per particle, *K*/*N*, peak at the critical temperature, *T*_c_, at the critical density *ρ*_c_. Error bars indicate the standard deviation (1*σ*) for parameter estimates from linear fits in **a**

Cooling the fluid towards the critical point, there are long wavelength fluctuations in density that cause the correlation length to diverge. In our simulations, though, the correlation length saturates at the size of our simulation cell, *L*, truncating longer wavelength fluctuations; only replicating periodic fluctuations through our boundary conditions may be a source of the shallowness of the minima in the largest Lyapunov exponents (Fig. 1b). Fleeting clusters of all sizes up to *L*, which will eventually become liquid, begin to appear. These clusters’ ephemeral existence affects instability in the critical dynamics on short timescales. As the structure evolves, the dynamics continues to temporarily sample phase space domains where trajectories diverge more quickly and more slowly than the average, domains that will determine the Lyapunov vector directions and the finite-time estimates of the Lyapunov exponents. To analyze the fluctuations in finite-time Lyapunov exponents *λ*_*i*_(*t*), we divided trajectories of 10^6^ time steps uniformly into windows of 100 time steps. Distributions of *λ*_1_(*t*) are shown in Fig. 3a.

Non-trivial^43^ power laws are apparent in the decay of finite-time Lyapunov exponent fluctuations with system size. At thermodynamic equilibrium, the precise scaling of relative fluctuations is often called self-averaging^44^. Loosely, a system is self-averaging with respect to a given property, *X*, if the value of the thermodynamic observable corresponds to the average over independent subsystems or, in this case, time windows. More precisely, the relative fluctuations of an observable *X* are $R_X = \left\langle {{\mathrm{\Delta }}X^2} \right\rangle /\left\langle X \right\rangle ^2\sim D_X/\left\langle X \right\rangle ^2\sim N^{ - \gamma }$ with Δ*X* = *X*−〈*X*〉, the wandering exponent *γ*, and a generalized diffusion coefficient *D*_*X*_. If the observable is self-averaging, the relative variance *R*_*X*_ of the property *X* vanishes in the thermodynamic limit: *R*_*X*_ → 0 when *N* → ∞. The wandering exponent *γ* can have values between 0 and 1—a value of one meaning the observable is strongly self-averaging. Weakly self-averaging observables have *γ*  < 1 and non-self-averaging observables have *γ* = 0.

Because of the statistical independence of spatial domains in the system, equilibrium observables are often strongly self-averaging with *γ* = 1. However, these domains become statistically dependent near a critical point because of the divergence of the correlation length^1^. Statistical signatures of dynamical observables, like the Lyapunov exponents, are still being elucidated^26,45^. Deep in the liquid state, for example, the fluctuations for all but the first Lyapunov exponent (the “bulk”) are strongly self-averaging. The first exponent fluctuations, however, self-average weakly with a rate of decay *γ*  < 1 that depends on the length scale of the interparticle interactions and captures the van der Waals picture of dominant repulsive forces^33^. How does this dynamical version of the van der Waals picture change at the critical point?

Numerical calculation of the entire Lyapunov spectrum comes at significant computational cost, cost that increases significantly when scaling with system size^46^. However, to quantify the scaling behavior of the finite-time Lyapunov exponent fluctuations with system size, we simulated 11 systems ranging from *N* = 100 to 2000 over the same range of temperatures at fixed density *ρ*_c_. Over this range of system sizes, fluctuations in the first finite-time Lyapunov exponent, *λ*_1_(*t*), as measured by the diffusion coefficient $D_{\lambda _1}(N,T,\rho )$, decay with system size as *N*^−*γ*^. This scaling holds over the temperature range *T* = 0.8–1.4 spanning the critical point (Fig. 3a). The wandering exponent varies between 0.9 and 0.6 (with its smallest value near *T*_c_), showing temperature controls the magnitude of the wandering exponent through the structural changes and spatial correlations it brings about near *T*_c_. A higher-order statistical analysis indicates this weak self-averaging is, at least in part, due to the non-Gaussian features of the distributions (Supplementary Note 2). Distributions of the kinetic energy per particle have a strong Gaussian character under all conditions we simulate.

Most prominent in the temperature dependence of the wandering exponent of the first exponent is the peak at *T*  = 0.962 (Fig. 3b). It shows the critical dynamics self-average most weakly in the direction of the first Lyapunov vector. As a reference, the fluctuations in kinetic energy per particle also decay with system size; the wandering exponent peaks at *T* = 0.937, further confirming the location of *T*_c_ and further quantifying the decay of global fluctuations (Fig. 3b). This critical temperature agrees with that found from grand canonical Monte Carlo simulations^34^. The peak in −*γ* for the first Lyapunov exponent is just above the critical temperature. However, the wandering exponent −*γ* and its maximum are affected by the range of system sizes and the statistical error in the linear fits of Fig. 3b. Near the maximum, −*γ* values appear to converge from below and, so, we take them as a lower bound (Supplementary Fig. 9).

**Fig. 4:**
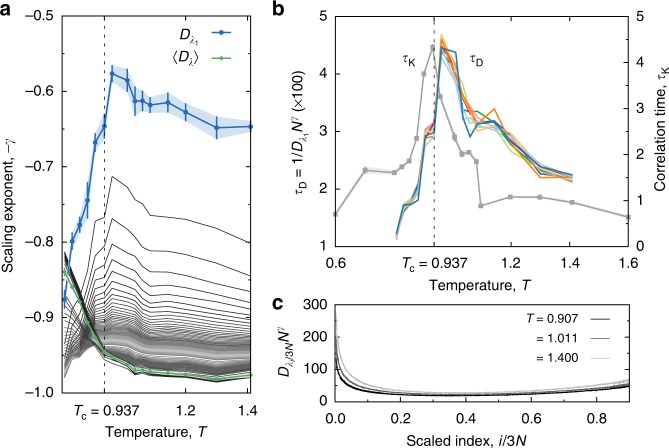
Maxima in dynamical timescales of Lyapunov exponent fluctuations. Weak self-averaging for the largest positive exponents indicates statistical dependence of spatial domains near the critical point. **a** The (negative) wandering exponent as a function of temperature *T* at *ρ*_c_ for the diffusion coefficient of the first finite-time Lyapunov exponent. Also shown are the −*γ* for average diffusion coefficients where averaging includes an increasing number of Lyapunov exponents in the spectrum. **b** Peak in the dynamical timescale for Lyapunov exponent fluctuations, $\tau _{\mathrm{{D}}} \equiv 1/D_{\lambda _1}N^\gamma$, and peak in the correlation time of kinetic energy per particle, *τ*_K_. **c** Data collapse for the scaled diffusion coefficient of finite-time Lyapunov exponent spectra as a function of the scaled spectral indices: the functional form is the scaling function $\tilde D(T,\rho = \rho _{\mathrm{c}})$. All data are at the critical density for the three-dimensional Lennard–Jones fluid. Error bars indicate the standard deviation (1*σ*)

The order of the thermodynamic limit and the time limit in the definition of the Lyapunov exponents can determine whether a dynamics is chaotic or not^45,47^. Because of the subtleties of the time and thermodynamic limit order, fluctuations in the finite-time Lyapunov exponents of both dissipative and conservative dynamical systems have recently been subject to a finite-size scaling analysis^26,33,45^. In all cases reported to date, the self-averaging of the first exponent is distinct from that of the bulk, the set of 3*N*−1 positive exponents that exclude the first. In spatially-extended dynamical systems where it is known, the scaling of fluctuations is homogeneous across the bulk of the spectrum^26,48,49^. Liquids show this behavior, for example, and all the bulk exponents are strongly self-averaging^33^. However, the critical dynamics break this scaling symmetry—a significant fraction of the bulk exponents self-average weakly as shown in Fig. 4. This scaling feature is so far unique to the liquid–vapor critical point. It is also apparent in the self-averaging of the entire Lyapunov spectrum through the average diffusion coefficient $\langle D(\lambda )\rangle = \mathop {\sum}\nolimits_{i = 1}^{6N} D (\lambda _i)/6N$, which contrasts that of the largest exponent (Fig. 4a). The corresponding wandering exponent has an inflection point around *T* = 0.962. Increasing the fraction of exponents included in the average shows that a portion of the more unstable vectors have a *γ*-peak that vanishes with increasing index.

While fluctuations appear to decay with system size in all unstable phase space directions on our accessible time and length scales, the rate of decay is far from homogeneous. The *γ*-spectrum quantitatively resolves the rates at which this unique thermodynamic equilibrium state emerges from the molecular dynamics (Fig. 4a). The good data collapse in Fig. 4c reveals clear scaling functions for the diffusion coefficient spectrum, $\tilde D(T,\rho = \rho _{\mathrm{c}})$; only three representative temperatures are shown. This scaling function is a system-size independent measure of the finite-time fluctuations in the Lyapunov spectrum, fluctuations caused by the local heterogeneities in phase space sampled by our simulated trajectories. The basic form of this scaling function is similar to that seen for simple liquids^33^ and Hamiltonian lattices^45^. Our calculation of $D_{\lambda _1}$, a dynamical invariant in the long time limit, leads directly to a dynamical timescale for *λ*_1_(*t*) fluctuations, $\tau _{\mathrm{{D}}} = 1/D_{\lambda _1}N^\gamma$. This timescale peaks just above the critical temperature^34^. Although these vectors are highly active on short timescales, the fluctuations destructively interfere on longer timescales (showing a net suppression, Fig. 1). The peak in the correlation time of the kinetic energy per particle is evidence of critical slowing down (Fig. 4b), and confirms the location of the critical temperature.

## Discussion

Based on early foundational work, there are known connections between hydrodynamics and Lyapunov vectors with small, but finite, exponents^50^. The dynamics of these so-called hydrodynamic modes can characterize macroscopic transport^36,51^. Here, above the liquid–vapor critical point, the Lyapunov exponents of these modes depend strongly on temperature but show no clear signs of the critical dynamics. Recently, though, it was found that hydrodynamics can shape the most unstable directions as well; numerical simulations of two prototypical Hamiltonian lattices, the FPU-*β* and the Φ_4_ models, demonstrated that long-range correlations can have a dramatic effect on the fluctuations of the leading finite-time Lyapunov exponent and even cause them to diverge with system size^45^. The minima we observe in the long-time exponents of fluids near *T*_c_ show the most unstable Lyapunov vectors are also sensitive to the correlations induced by critical phenomena.

Dynamical systems have begun to collect into universality classes through the finite-size scaling of Lyapunov exponent fluctuations. Numerical estimates of the wandering exponent *γ* reported^26,48,49^ so far in the literature suggest the Lyapunov exponents of dissipative systems are weakly self-averaging, *γ* < 1, with the dynamics of the first Lyapunov vector belonging to the Kardar–Parisi–Zhang (KPZ) universality class^52^. Fluctuations of the maximum Lyapunov exponent in Hamiltonian lattice models, FPU-*β* and the Φ_4_, however, are not self-averaging and each belong to their own universality class^45,53^. There is mounting evidence that their non-KPZ behavior is a consequence of long-range spatiotemporal correlations and not a more universal feature of Hamiltonian dynamics^33^. The weakening of the self-averaging behavior and peak in −*γ* near the liquid–vapor critical point is further support for this hypothesis. Because the molecular dynamics at the liquid–vapor critical point and in the liquid state^33^ are Hamiltonian, however, the dynamic universality class of the Lyapunov vector dynamics remains an open question.

Though distinct from the fluids here, models for non-additive systems with long-range (gravitational or electrostatic) forces have shed some light on the relationship between microscopic chaos at phase transitions. The dynamics of the Hamiltonian mean-field model, for example, are chaotic for finite-size systems but not for an infinite size system^47^. The dynamics and thermodynamics that have been studied both numerically^54–57^ and analytically^58^. And, in contrast to the results here, near the second-order phase transition, both the largest Lyapunov exponent and kinetic energy fluctuations have a maximum near the critical temperature^55^.

Here, by treating the nonlinear dynamics directly with numerical simulations and analytical models, we have resolved the phase space directions responsible for the thermodynamic instability and the breakdown of the van der Waals picture at the liquid–vapor critical point. The long-time Lyapunov spectra show that critical dynamics of finite-size fluidic systems are less sensitive to the detailed features of intermolecular forces but also initial conditions. Through numerical simulations and analytical models, critical conditions appear to constrain the dynamics so that different phase space directions have a relatively homogeneous degree of instability and scaling features in finite-time Lyapunov exponent fluctuations that are so far unique to critical dynamics.

Correlations in molecular positions at the critical point span the length scales of intermolecular forces to the entire system. They imply structural organization that is intrinsically opposed by the chaotic dynamics. The mechanisms balancing this internal tension between order and dynamical instability, however, are subtle. As a result, theoretical explanations for the mechanical origins of critical phenomena are uncommon. Continuous transitions in crystals are a notable exception, where structural changes arise through the instability of a lattice vibration^9^. There, the mode responsible for the phase transition is a collective excitation whose frequency decreases anomalously during an approach to the transition point. For example, in SrTiO_3_ the frequency of a soft phonon mode decreases substantially and, ultimately, freezes at the transition temperature when approached from below^59^.

Unlike continuous crystal–crystal phase transitions, there is not one unique unstable vector with a vanishing frequency at the liquid–vapor critical point. Instead, the whole spectrum softens with a subset that have extrema near the critical point; both the long-time Lyapunov exponents and their fluctuations on short times reflect their high sensitivity to long-range correlations of molecular positions. These vectors do not appear to completely freeze, at least not in finite-size systems, but do exhibit large fluctuations with a peak in dynamical timescales indicating the critical slowing down of chaos, the stabilization of unstable vectors, and a longer memory of initial conditions. In short, the relative mechanical stability of molecular motion underlies the bulk behavior of fluids at this thermodynamic instability.

## Methods

### Model non-associated fluid

Our system is the three-dimensional, periodic Lennard–Jones (LJ) fluid^29^. The Hamiltonian of this collection of *N* particles is $H(r_{ij},p_k) = \mathop {\sum}\nolimits_k^{3N} {p_k^2} /2m + \mathop {\sum}\nolimits_{i < j}^N V (r_{ij})$. The pairwise interaction potential between particles *i* and *j* a distance $\tilde r_{ij}$ apart is given by

2 $$V(\tilde r_{ij}) = 4\epsilon _{ij}\left[ {\left( {\frac{\sigma }{{\tilde r_{ij}}}} \right)^{12} - \left( {\frac{\sigma }{{\tilde r_{ij}}}} \right)^6} \right].$$

The parameter *ε*_*ij*_ corresponds to the well depth at the equilibrium distance and measures the strength of the interaction between particles *i* and *j*. For the single-component fluid we consider, all interactions are identical (*ε*_*ij*_ = *ε*). The first term of the potential energy function takes into account short-range repulsive interactions. The second term corresponds to the attractive part of the interaction, which acts over a comparatively longer range. The parameter *σ* stands for the distance at which the attractive and the repulsive forces are equal and can serve as a measure of the particle size. We work in LJ reduced units with the distance $r = \tilde r/\sigma$, density $\rho = \tilde \rho \sigma ^3$, temperature $T = k_{\mathrm{{B}}}\tilde T/\varepsilon$, time $t = \tilde t/\sqrt {m\sigma ^2/\varepsilon }$, and Lyapunov exponents $\lambda = \tilde \lambda \sqrt {m\sigma ^2/\varepsilon }$. All particles have unit mass *m* = 1, *ε* = 1, and *σ* = 1.

### Numerical simulation methods

All numerical data are from molecular dynamics simulations of constant energy trajectories. The initial conditions were sampled from a constant temperature trajectory using the Berendsen thermostat with a time constant of 0.5. Subsequent *NVE* trajectories are updated with the velocity Verlet algorithm using a time step of 10^−3^. Their total energy is well conserved and the kinetic temperature fluctuates around the temperature of the sampled *NVT* trajectory. We shift and truncate the interparticle potential^60^ and use a Verlet list with a 2.5*σ* cutoff and a “skin” of 0.1*σ*. All data are from single-precision calculations.

At fixed number of particles *N*, volume *V*, and energy *E*, we simulate deterministic trajectories of this equilibrium fluid and the dynamics of Lyapunov vectors in tangent space^46^. The *i*th Lyapunov vector components are first variations in position and momentum (*δq*_*ij*_, *δp*_*ij*_)^T^ with *i*, *j* = 1, …, 6*N* and evolve according to their own linearized Hamiltonian equation of motion. We numerically solve this equation of motion with the linearized form of the velocity Verlet algorithm used to evolve trajectories and orthonormalization at every time step^46^. The initial basis sets are random and orthonormal. During a transient, that we discard, the first vector orients itself parallel to the maximally changing tangent space direction. Regular orthonormalization restricts the collapse of the remaining vectors onto the most expanding tangent space direction. The algorithm requires the second derivatives (Hessian) of the interaction potential at every time steps. We use forward differences of the analytical gradients with a displacement of 10^−4^.

Within the linearized limit, the expansion or the compression factor along the phase-space direction of the *i*th Lyapunov vector over time *t* is ${\mathrm{{e}}}^{\Gamma _i(t)}$. The corresponding finite-time Lyapunov exponent is *λ*_*i*_(*t*) = Γ_*i*_(*t*)/*t*. The complete finite-time Lyapunov spectrum, {*λ*_*i*_(*t*)} is calculated from the set of Gram–Schmidt vectors with standard methods^61,62^. We evaluate the full Lyapunov spectrum at each time step using the norm $\left| {\delta x_i(t^\prime )} \right| = \left[ {\mathop {\sum}\nolimits_j^{6N} \delta q_{ij}(t^\prime )^2 + \delta p_{ij}(t^\prime )^2} \right]^{1/2}$. The *i*th finite-time exponent over a time interval *t* = *t*′−*t*_0_ has the form:

3 $$\lambda _i(t) = \left| {t^{\prime} - t_0} \right|^{ - 1}{\mathrm{ln}}\frac{{\left| {\delta x_i(t^{\prime})} \right|}}{{\left| {\delta x_i(t_0)} \right|}}.$$

At each thermodynamic condition, simulations of long trajectories are 10^6^ time steps and span a time of *t*_final_ = 1000 in reduced time units. For each long trajectory we obtain estimates of the long-time exponents {*λ*_*i*_}.

Each trajectory is also used to calculate the finite-time Lyapunov exponents by dividing the long trajectory into constant time intervals of size of 0.1. Every time segment has a unique initial condition, so the partitioning of the long-time trajectory produces an ensemble of 10,000 finite-time trajectories. Finite-time Lyapunov exponents are fluctuating variables. We estimate the magnitude of their fluctuations over a time interval, *t*, with the diffusion coefficients {*D*(*λ*_*i*_)}^26,33,45^ and the variance, $\chi _i^2(t)$, of {Γ_*i*_(*t*)}

4 $$tD(\lambda _i) = \chi _i^2(t) = \left\langle {\left( {\Gamma _i(t) - \left\langle {\lambda _i} \right\rangle t} \right)^2} \right\rangle .$$

Averages 〈⋅〉 are over an ensemble of 10^4^ trajectories, each having time span *t* = 0.1 in reduced time units. The average 〈*λ*_*i*_〉 = *λ*_*i*_ is the average of the *i*th Lyapunov exponent over an entire long trajectory. While the finite-time Lyapunov exponents depend on the chosen norm, the diffusion coefficient is a dynamical invariant^45^.

To probe the self-averaging property of finite-time Lyapunov exponent fluctuations, we ran trajectories over a range of system sizes. From *N* = 100 up to *N* = 2000, we calculated the diffusion coefficient of the finite-time {*λ*_*i*_(*t*)} over the trajectory ensemble for each system size. We scaled the number of molecules *N* and the volume *V* to ensure the thermodynamic limit of the microcanonical ensemble: *N*, *V* → ∞ keeping the number density *ρ* = *N*/*V* and energy density *e* = *E*/*V* constant. According to the equipartition theorem, the kinetic temperature of the system is given by *T* = 2〈*E*_kin_〉/3*Nk*_B_. We analyze the scaling of fluctuations in dynamical variables with system-size *N* for a range of *T* with *ρ* fixed at *ρ*_c_ = 0.317. From the scaling of {*D*(*λ*_*i*_)} with *N*, we estimate the values of the wandering exponents *γ*. Estimates of the wandering exponent with up to *N* = 1000 or *N* = 2000 particles does not affect the location of maximum in −*γ* or qualitative features of its temperature dependence, suggesting our estimates are well converged. The power-law decay of the diffusion coefficient of the finite-time Lyapunov exponents with system size was also found to be robust to changes in the chosen time interval.

### Analytical model of nonlinearly coupled oscillators

We consider a one-dimensional system of *N* interacting, classical particles of mass *m* in a bistable potential $V(x_i) = Ax_i^4 + Bx_i^2$. The single-particle Hamiltonian is

5 $$H_1(x_i,p_i) = \frac{{p_i^2}}{{2m}} + Ax_i^4 + Bx_i^2 + \mathop {\sum}\limits_{\left\langle {ij} \right\rangle } {Jx_ix_j}$$

where 〈*ij*〉 indicates all pairwise particle interactions with interaction strength *J*. Here, we use *A* = *κ*_4_/4 and *B* = −*κ*_2_/2 with positive constants *κ*_2_, *κ*_4_ > 0. We drop the particle index in what follows. The minima of the potential wells are at *x* = ±*x*_0_ with *x*_0_ = *κ*_2_/*κ*_4_ with potential energy $V_0 = - {\textstyle{1 \over 4}}\kappa _2x_0^2$. The interaction energy for particles at their minima is $Jx_0^2$. The Ising limit is when the barrier separating the two wells is high relative to the interaction energy, $|V_0|/Jx_0^2 \gg 1$, so that each particle is localized in the left (0 state) or right (1 state) well. Particles may randomly occupy these 0 and 1 states.

Two approximations of this model capture the singularity and the finite-jump discontinuity of the isochoric heat capacity at the liquid–vapor critical point. Both approximations decouple the oscillators. Let *δx*_*i*_ = (*δq*_*i*_, *δp*_*i*_) be a variation of a single-particle phase point. Its equation of motion is

6 $$\delta \dot x_i = {\mathrm{\Omega }}D^2H_1\delta x_i$$

where Ω is the symplectic matrix, *D*^2^*H*_1_ is the second derivative of the relevant single-particle Hamiltonian, and

7 $${\mathrm{\Omega }}D^2H_1 = \left( {\begin{array}{*{20}{c}} 0 & T \\ { - D_x^2H_1} & 0 \end{array}} \right).$$

The submatrix *T* is diag(1/*m*) and $D_x^2H_1$ is the Hessian matrix. The eigenvalues of Ω*D*^2^*H*_1_ satisfying the characteristic polynomial $\lambda ^2 + m^{ - 1}D_x^2H_1 = 0$ are the local Lyapunov exponents $\lambda _ \pm = \pm {\mathrm{Re}}\sqrt { - D_x^2H_1/m}$. In both approximations below, each particle contributes one Lyapunov exponent. As a result, the Lyapunov spectrum and the Kolmogorov–Sinai entropy are system-size extensive, *h*_KS_ = *Nλ*_+_.

Soft-mode approximation: To analyze the effect of a diverging heat capacity on the Lyapunov exponents, we treat the average effects of the anharmonic term in Eq. (5) by imposing a temperature-dependent weakening of the anharmonic restoring force on each particle^9^. We set *J* = 0 and *x*^4^ → *x*^2^〈*x*^2^〉_0_, using the canonical average of the displacement squared for the harmonic oscillator 〈*x*^2^〉_0_ = *k*_B_*T*/*κ*_2_. The Hamiltonian of a single particle becomes

8 $$H_1^\prime (x,p) = \frac{{p^2}}{{2m}} + \left( {A\left\langle {x^2} \right\rangle _0 + B} \right)x^2.$$

The critical temperature is $k_{\mathrm{B}}T_{\mathrm{c}} = 2\kappa _2^2/\kappa _4$, which we can use to express the free energy of a single particle

9 $$\beta F_1(T) = \beta F_{0,1}(T) - {\mathrm{ln}}\sqrt {\frac{{T_{\mathrm{c}}}}{{T - T_{\mathrm{c}}}}}$$

for *T* > *T*_c_ with the free energy of a single harmonic oscillator *F*_0,1_. Using the thermodynamic relation *C*_*V*_ = −*T*∂^2^*F*/∂*T*^2^, the isochoric heat capacity of each particle is

10 $$C_{V,1} = k_{\mathrm{B}} - k_{\mathrm{B}}T\frac{{T - 2T_{\mathrm{c}}}}{{2\left( {T - T_{\mathrm{c}}} \right)^2}}$$

and *C*_*V*,1_ → ∞ as *T* → *T*_c_ from above.

The canonical ensemble average Lyapunov exponents are

11 $$\lambda _ \pm = \left\langle {\lambda _ \pm } \right\rangle = {\int\nolimits_{ - \infty }^{\infty}} {\int\nolimits_{ - \infty }^{\infty}} {\lambda _ \pm } (x)\,\rho (x,p)\,{\mathrm{d}}x\,{\mathrm{d}}p$$

where $\rho (x,p) = Z^{ - 1}{\mathrm{e}}^{ - \beta H_1^\prime (x,p)}$ is the canonical probability density with partition function $Z(\beta ) = {\int}_{ - \infty }^\infty {\int}_{ - \infty }^\infty {\mathrm{e}}^{ - \beta H_1^\prime (x,p)}\,{\mathrm{d}}x\,{\mathrm{d}}p$. Because the local exponents do not depend on position *x*, the global exponents are explicitly given by

12 $$\left\langle {\lambda _ \pm } \right\rangle = \lambda _ \pm (T) = \pm \sqrt {\frac{{\kappa _2}}{m}} \left( {\frac{{|T - T_{\mathrm{c}}|}}{{T_{\mathrm{c}}}}} \right)^{1/2}.$$

If the space and time averages are equivalent, then $\lambda _\pm = \left\langle {\lambda _\pm} \right\rangle = {\mathrm{lim}}_{t \to \infty }\lambda _\pm(t)$. The right eigenvectors of ${\mathrm{\Omega }}D^2H_1^\prime$ are (1/*m*, *λ*_+_)^T^ and (1/*m*, −*λ*_+_)^T^. At the critical point, the Lyapunov exponents are degenerate and these eigenvectors are linearly dependent. Notice that because *λ*_±_ = *λ*_±_(*x*), there are no spatial fluctuations: $\left\langle {\lambda _ \pm ^2} \right\rangle - \left\langle {\lambda _ \pm } \right\rangle ^2 = 0$. The precise form of the critical exponent will depend on the nature of the correlations near the transition temperature. While this approximation neglects these correlations, the heat capacity does diverge at *T*_c_, which is also a feature of the liquid–vapor critical point, with a concomitant divergence in the Lyapunov time and degeneracy of the Lyapunov spectrum.

Mean-field approximation: Applying a mean-field approximation to the original Hamiltonian^40–42^, leads to an isochoric heat capacity with a finite-jump discontinuity (Supplementary Fig. 7). We replace the bilinear interaction between particles by −*J* 〈*x*〉 *x*, where 〈*x*〉 is the ensemble average displacement. The addition of *J* 〈*x*〉^2^/2 avoids double counting interactions. The Hamiltonian of a single particle is then

13 $$H_{{\mathrm{MF}}} = \frac{{p^2}}{{2m}} + V(x) - J\left\langle x \right\rangle x + {\textstyle{1 \over 2}}J\left\langle x \right\rangle ^2.$$

For our choice of *A* = *κ*_4_/4 and *B* = −*κ*_2_/2, the potential *V*(*x*) is a double well. We neglect the kinetic energy in what follows. The equilibrium free energy of each particle is

14 $$F_{{\mathrm{MF}}}(T) = - k_{\mathrm{B}}T{\mathrm{lnTr}}[\rho _{{\mathrm{MF}}}(x)] + {\textstyle{1 \over 2}}J\left\langle x \right\rangle ^2$$

where *ρ*_MF_(*x*) = exp{−*βV*(*x*) + *βJ*〈*x*〉*x*} and Tr[⋅] is the configuration integral. The free energy is bistable for *T* < *T*_c_ and monostable for *T* ≥ *T*_c_. Taking 〈*x*〉 to be a free parameter, the minimum of the free energy at

15 $$\frac{{\partial F_{{\mathrm{MF}}}\left( {T,\left\langle x \right\rangle } \right)}}{{\partial \left\langle x \right\rangle }} = 0$$

gives a constraint on the possible microstates consistent with this macrostate. Using the mean displacement as an order parameter, subject to the constraint above, the free energy is a minimum only when 〈*x*〉 satisfies the self-consistency condition

16 $$\left\langle x \right\rangle = \frac{{{\mathrm{Tr}}[x\rho _{{\mathrm{MF}}}(x)]}}{{{\mathrm{Tr}}[\rho _{{\mathrm{MF}}}(x)]}}.$$

The minimum value *M* is the mean displacement of a particle at equilibrium. This order parameter is nonzero when *T* < *T*_c_ (the ordered phase) and is zero when *T* ≥ *T*_c_ (the disordered phase).

The critical temperature is found by making contact with the Landau free energy for continuous phase transitions. At equilibrium, the free energy becomes $F_{{\mathrm{MF}}}(T,M) = - k_{\mathrm{B}}T{\mathrm{lnTr}}[\rho _{{\mathrm{MF}}}(x)] + {\textstyle{1 \over 2}}JM^2$. We consider symmetric potential functions *V*(*x*) = *V*(−*x*) so that only even terms survive in the cumulant expansion

17 $${\mathrm{ln}}\left\langle {{\mathrm{e}}^x} \right\rangle _0 = \mathop {\sum}\limits_{n = 1}^\infty {\frac{{\left\langle {x^n} \right\rangle _0}}{{n!}}}$$

where 〈*O*〉_0_ represents Tr[*O*(*x*)*ρ*_0_(*x*)]/Tr[*ρ*_0_(*x*)] and *ρ*_0_(*x*) = e^−*βV*(*x*)^. Up to second order, the expanded free energy is

18 $$F_{{\mathrm{MF}}}(T,M) = F_0(T) + {\textstyle{1 \over 2}}JM^2\left( {1 - \beta J\left\langle {x^2} \right\rangle _0} \right).$$

The second term vanishes at the critical temperature *k*_B_*T*_c_ = *J* 〈*x*^2^〉_0_ (Supplementary Fig. 6).

Taylor expanding the coefficient of the second term in the free energy when *T* is near *T*_c_ gives an expression that agrees with the Landau free energy to second order

19 $$F_{{\mathrm{MF}}}(T,M) = F_0(T) + {\textstyle{1 \over 2}}M^2\left( {J - \frac{{J^2}}{{k_{\mathrm{B}}}}\frac{{{\mathrm{d}}\left\langle {x^2} \right\rangle _0}}{{{\mathrm{d}}T}}} \right)\frac{{\left( {T - T_{\mathrm{c}}} \right)}}{{T_{\mathrm{c}}}}.$$

This expression does not explicitly use the form of the potential *V*(*x*), only that it is symmetric^42^. At a given *β* = 1/*k*_B_*T*, we self-consistently solve for *M* to find the optimal approximation of the original Hamiltonian. The canonical ensemble average of each observable *O* is Tr[*Oρ*_MF_(*x*)]/Tr[*ρ*_MF_(*x*)] with 〈*x*〉 = *M*. Assuming ergodicity, the ensemble average Lyapunov exponent *λ*_±_ = 〈*λ*_±_〉 is equal to the time average ${\mathrm{lim}}_{t \to \infty }\lambda _\pm(t)$.

The susceptibility diverges at the critical point (Supplementary Fig. 7). Following a standard procedure in Landau–Ginzburg theory^63^, the order parameter is coupled to an external field, *ξ*. In this case, the minimization of the free energy

20 $$\tilde F_{{\mathrm{MF}}}(T,M,\xi ) = F_{{\mathrm{MF}}}(T,M) - \xi M.$$

determines the equilibrium value *M*. We see that $\partial \tilde F_{{\mathrm{MF}}}/\partial M = 0$ when $JM\left( {1 - \beta J\left\langle {x^2} \right\rangle _0} \right) = \xi$. From this expression, the inverse susceptibility is

21 $$\chi ^{ - 1} = \frac{{\partial \xi }}{{\partial M}} = J\left( {1 - \beta J\left\langle {x^2} \right\rangle _0} \right)$$

and *χ*^−1^ → 0 as *T* → *T*_c_.

## Supplementary information

Supplementary Information

## Data Availability

The data are available from the corresponding author upon request.
