# Supplementary material for: Critical fluctuations and slowing down of chaos
Source: Nat Commun. 2019 May 14;10:2155. doi: 10.1038/s41467-019-10040-3 (PMC6517405; doi:10.1038/s41467-019-10040-3)
Supplement: Supplementary file 1 — Supplementary Information [file 41467_2019_10040_MOESM1_ESM.pdf]

Supplementary Information:
Critical fluctuations and slowing down of chaos

Moupriya Das[1] and Jason R. Green[1,2,3,*]

[1] *Department of Chemistry, University of Massachusetts Boston, Boston, MA 02125*
[2] *Department of Physics, University of Massachusetts Boston, Boston, MA 02125*
[3] *Center for Quantum and Nonequilibrium Systems, University of Massachusetts Boston, Boston, MA 02125*

[*] jason.green@umb.edu

SUPPLEMENTARY NOTE 1

To quantify slowing down in the critical dynamics and confirm the location of the critical temperature, T_c, we calculate the autocorrelation function for the kinetic energy. We anticipate the weak self-averaging of the Lyapunov exponents and kinetic energy per particle to originate from the statistical correlations near the critical point. From measurements of the kinetic energies $\{Y_1, Y_2, \ldots, Y_N\}$ at time $\{t_1, t_2, \ldots, t_N\}$, the k-th lag autocorrelation coefficient is

$$C_k = \frac{\sum_{i=1}^{N}(Y_i - \langle Y \rangle)(Y_{i+k} - \langle Y \rangle)}{\sum_{i=1}^{N}(Y_i - \langle Y \rangle)^2}. \tag{1}$$

These coefficients are normalized and can have both positive and negative values. A greater magnitude of C_k implies stronger correlations. A value close to 0 indicates the data are uncorrelated. We define the autocorrelation coefficient at time lag Δt as $C(\Delta t)$. Supplementary Fig. 1 shows that the kinetic energy exhibits significant correlations that decay with the lag time. For the kinetic energy, $C(\Delta t)$ has significant values for small time lags and decays with increasing lag. The decay is modeled by an exponential function $Ae^{-\Delta t/\tau}$ with a constant A and characteristic time τ. Because of critical slowing down, this time peaks at the critical temperature and density, Supplementary Fig. 1b and Fig. 4 in the main text. This result indicates the presence of strong temporal correlations in the kinetic energy and confirms the location of the critical temperature $T_c = 0.937$. Further support comes from the peak in the value of the autocorrelation coefficient at the first lag with $\Delta t = 0.1$, $C_1(\Delta t = 0.1)$, which also peaks at the critical temperature, Supplementary Fig. 1c.

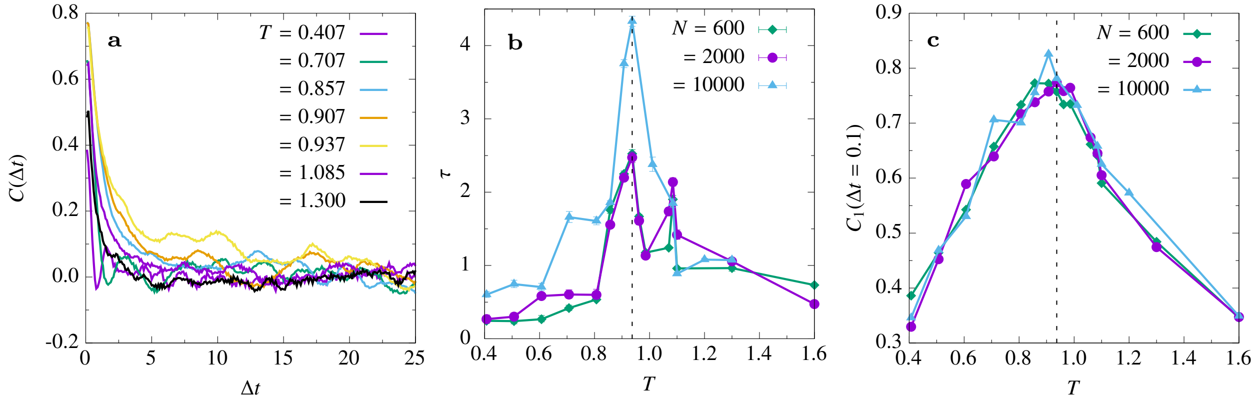

Supplementary Figure 1. **a** Autocorrelation coefficient $C(\Delta t)$ for the kinetic energy with time lag Δt at several temperatures at the critical density ρ_c for the three-dimensional $N = 600$ Lennard-Jones fluid. The **b** characteristic time of the kinetic energy autocorrelations and **c** autocorrelation coefficient at the first lag $C_1(\Delta t)$ for the kinetic energy, both as a function of temperature at the critical density ρ_c for $N = 600$, 2000, and $10,000$.

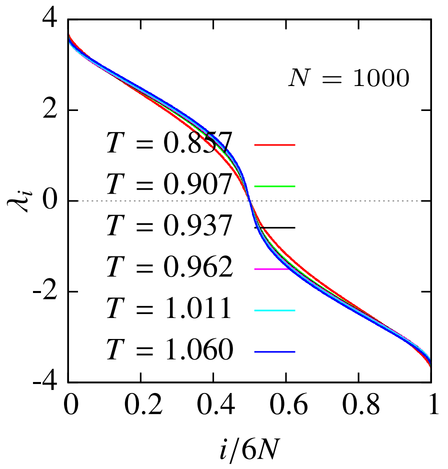

Supplementary Figure 2. Lyapunov spectra $\{\lambda_i\}$ with rescaled indices $i/6N$ for the three-dimensional Lennard-Jones fluid. At ρ_c, the spectral features are similar, but the positive area under the curve–the Kolmogorov-Sinai entropy–increases with increasing temperature.

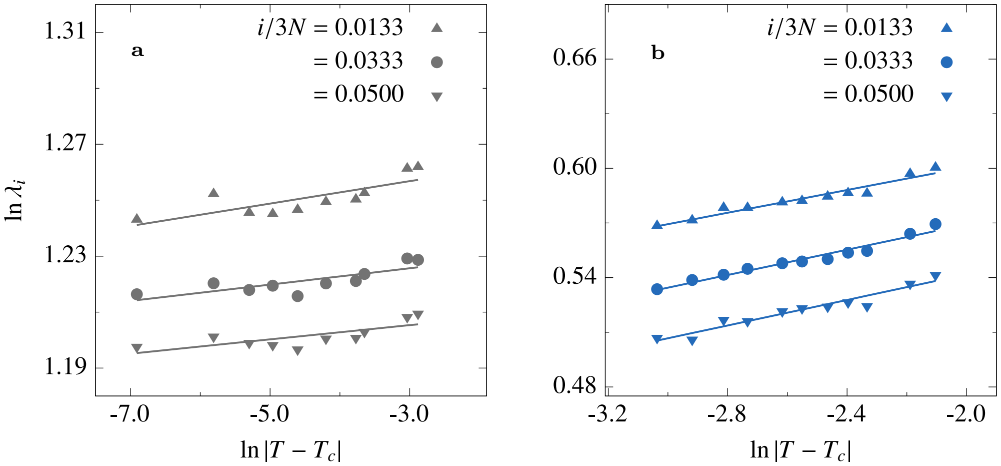

Supplementary Figure 3. Scaling of Lyapunov exponents near the critical temperature. **a**, **b** Representative scaling of the Lyapunov exponents as a function of $|T - T_c|$ (log-log) in the coexistence and supercritical regions using $T_c = 0.962$. Lines are best fits to the power law $\lambda_i \propto |T - T_c|^\alpha$. Fig. 1b of the main text shows that minima appear in the Lyapunov exponents of the most unstable modes (up to $i/3N \approx 0.18$) at $T = 0.962$ and ρ_c.

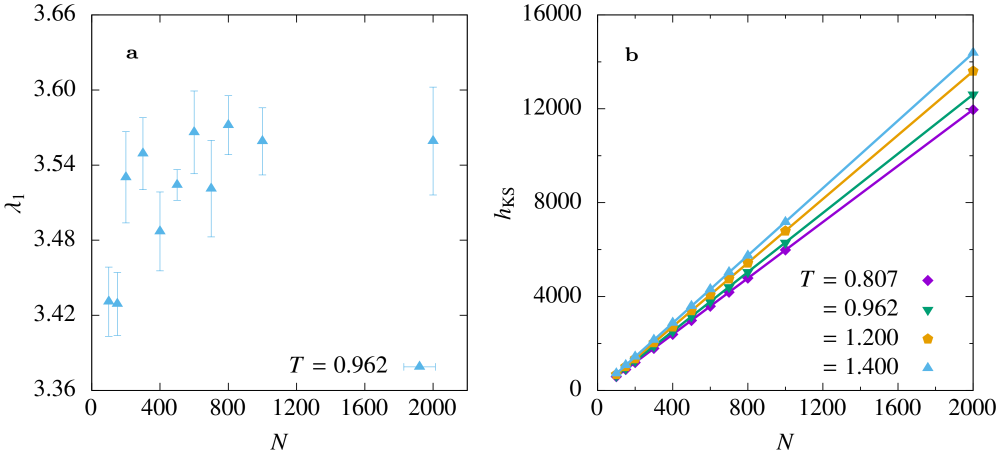

Supplementary Figure 4. Non-intensivity of the first Lyapunov exponent and extensivity of the Kolmogorov-Sinai entropy. **a** First Lyapunov exponent λ_1 as a function of system size N at the critical density $\rho_c = 0.317$ and temperature $T = 0.962$ for the three-dimensional Lennard-Jones fluid. **b** Linear scaling of the Kolmogorov-Sinai entropy with system size N at ρ_c at four representative temperatures. Contrary to the liquid state, Lyapunov exponents at the critical density do depend very weakly on the system size. As in the liquid state, the sum of the positive Lyapunov exponents, the Kolmogorov-Sinai entropy, increases linearly with system size. From additional simulations, we find the non-intensivity of Lyapunov exponents to be characteristic of the low density of the fluid, not just the critical density.

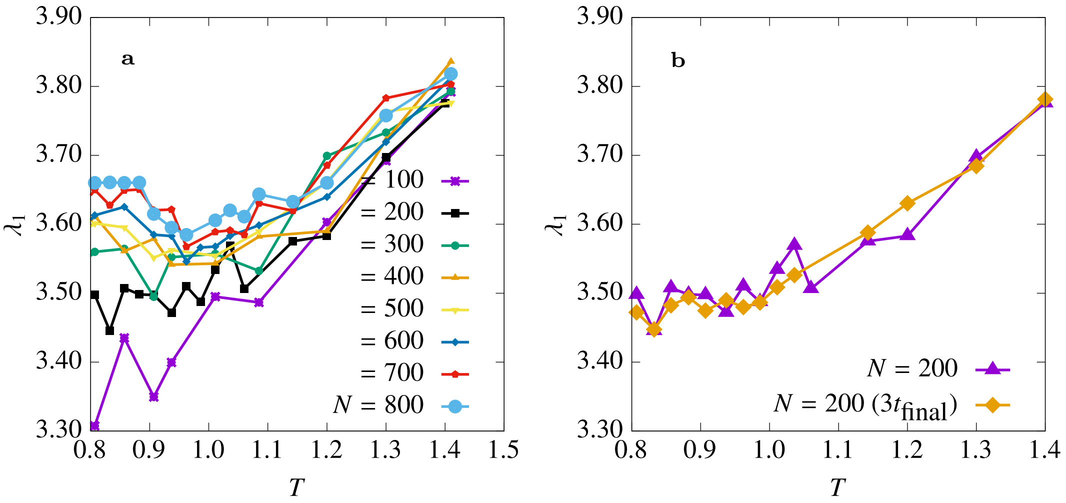

Supplementary Figure 5. **a** Largest Lyapunov exponent λ_1 versus temperature T for the three-dimensional Lennard-Jones fluid at ρ_c with $N = 100 - 800$. The value of the largest exponent at the minimum is 6-12% less than that at the highest T in supercritcial regime. The dependence of λ_1 on N has small effects on the temperature dependence and the location of the minimum (Supplementary Fig. 5) but mostly in the coexistence regime $T \leq T_c$. **b** Largest Lyapunov exponent λ_1 versus temperature T for two different total simulation times at ρ_c for the $N = 200$ three-dimensional Lennard-Jones fluid.

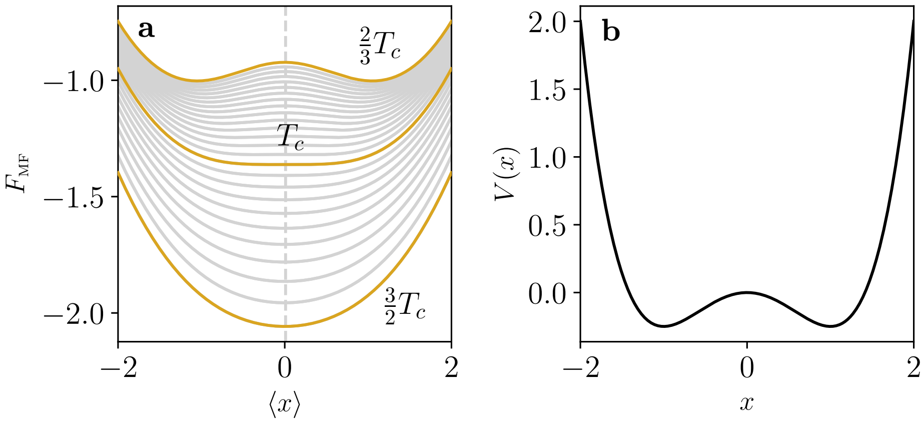

Supplementary Figure 6. Mean-field analysis of system of coupled nonlinear oscillators. **a** Mean-field free energy F_{MF} of coupled anharmonic oscillators in **b** the double-well potential over a range of temperatures from $T = \frac{2}{3}T_{\mathrm{c}}$ to $T = \frac{3}{2}T_{\mathrm{c}}$ spanning the critical temperature $T_{\mathrm{c}} = 1.0$.

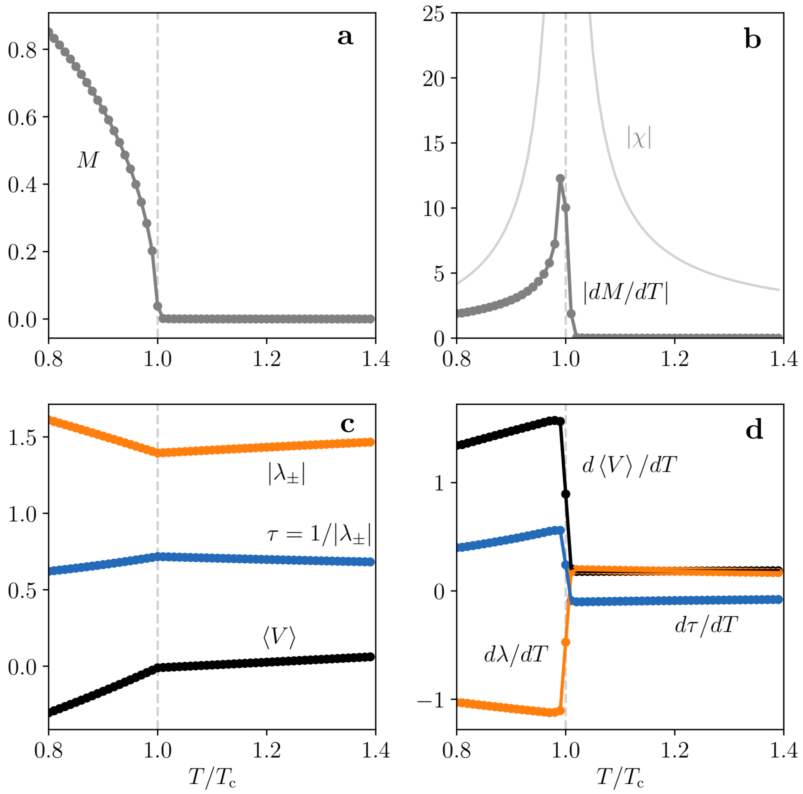

Supplementary Figure 7. Mean-field analysis of system of coupled nonlinear oscillators. **a** Order parameter, the mean particle displacement, $\langle x \rangle = M$, as a function of temperature. **b** The divergence of the susceptibility χ at the critical temperature T_{c} and the temperature derivative of the order parameter. **c** The Lyapunov exponent $|\lambda_\pm| = \langle|\lambda_\pm|\rangle$, Lyapunov time $\tau = 1/|\lambda_\pm|$, and mean potential energy $\langle V \rangle$ have a kink at the critical temperature; **d** their derivatives have a jump discontinuity at T_{c} (vertical dashed lines). The susceptibility $|\chi|$ also diverges at T_{c}.

SUPPLEMENTARY NOTE 2

The wandering exponent corresponding to the first Lyapunov exponent is always less than one over the temperature range studied. For the bulk of the spectrum, though, the wandering exponent is close to one above the critical temperature $T_\mathrm{c} = 0.937$. Strong self-averaging is expected for Gaussian-distributed variables. To test the hypothesis that the distribution of the finite-time, leading Lyapunov exponent has non-Gaussian features, we calculate the non-Gaussian parameter[1,2]. For an observable Y, this parameter is:

$$\alpha = \frac{\langle (Y - \langle Y \rangle)^4 \rangle}{5\langle (Y - \langle Y \rangle)^2 \rangle} - \frac{3}{5}. \tag{2}$$

The value of α is zero if the distribution is Gaussian. We calculated α for the entire Lyapunov spectrum for the $N = 700$ particle system at several temperatures and ρ_c. Six representative temperatures up to $i = 3N$ are shown in Supplementary Fig. 8. The parameter indicates that the distributions of largest positive Lyapunov exponents $\lambda_1(t)$ have significant non-Gaussian character that vanishes moving deeper into the bulk. These deviations from the normal distribution are at least partly responsible for the weak self-averaging of the first exponent. The distributions for the bulk of the spectrum, however, are roughly Gaussian and, consequently, self-average strongly.

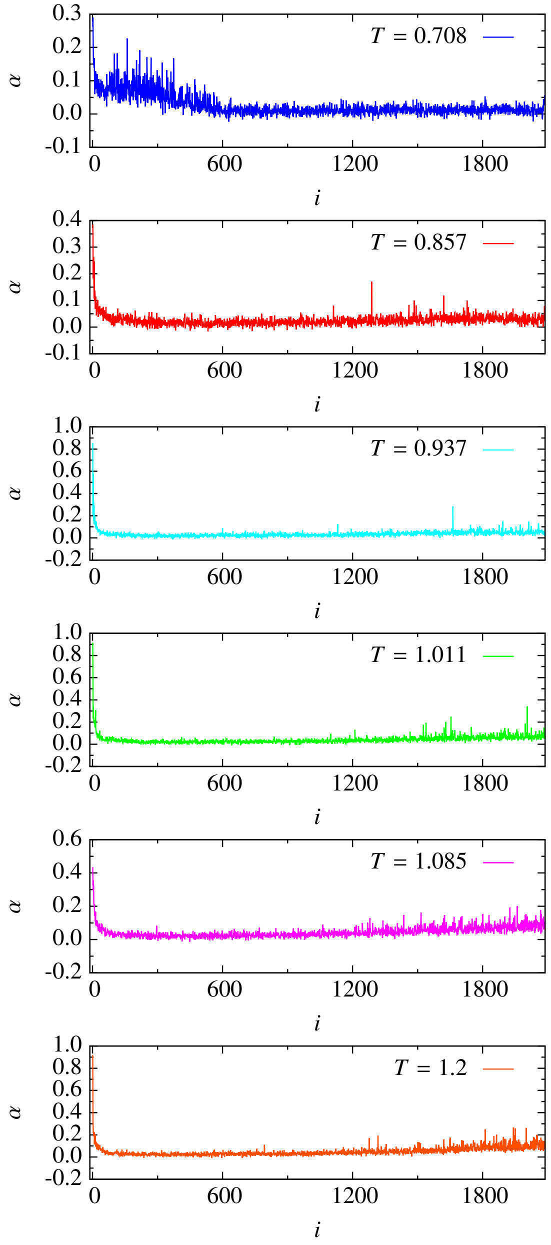

Supplementary Figure 8. Non-Gaussian parameter α versus the spectral index i at several temperatures and ρ_c for the $N = 700$ three-dimensional Lennard-Jones fluid.

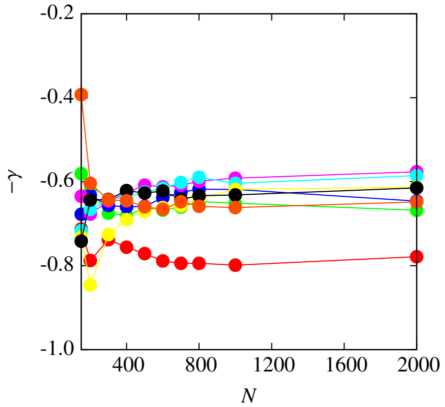

Supplementary Figure 9. Negative of wandering exponent $-\gamma$ as a function of system size N at different temperatures and ρ_c for the three-dimensional Lennard-Jones fluid. [Red: $T = 0.8325$, Green: $T = 0.907$, Blue: $T = 0.937$, Pink: $T = 0.962$, Cyan: $T = 1.011$, Yellow: $T = 1.036$, Black: $T = 1.1425$, Orange: $T = 1.3$]

SUPPLEMENTARY REFERENCES

[1] A. Rahman, "Correlations in the motion of atoms in liquid argon," Phys. Rev. **136**, A405 (1964).

[2] M. S. Shell, P. G Debenedetti, and F. H. Stillinger, "Dynamic heterogeneity and non-Gaussian behaviour in a model supercooled liquid," J. Phys. Condens. Matter **17**, S4035 (2005).
